# Rheumatic Immune-Related Adverse Events—A Consequence of Immune Checkpoint Inhibitor Therapy

**DOI:** 10.3390/biology10060561

**Published:** 2021-06-20

**Authors:** Anca Bobircă, Florin Bobircă, Ioan Ancuta, Alesandra Florescu, Vlad Pădureanu, Dan Nicolae Florescu, Rodica Pădureanu, Anca Florescu, Anca Emanuela Mușetescu

**Affiliations:** 1 Department of Internal Medicine and Rheumatology, Carol Davila University of Medicine and Pharmacy, 050474 Bucharest, Romania; anca.bobirca@umfcd.ro (A.B.); ioan.ancuta@umfcd.ro (I.A.); 2 Department of General Surgery, Carol Davila University of Medicine and Pharmacy, 050474 Bucharest, Romania; florin.bobirca@umfcd.ro; 3 Department of Rheumatology, Emergency Clinical County Hospital of Craiova, 200642 Craiova, Romania; alesandracioroianu@yahoo.com; 4 Department of Internal Medicine, University of Medicine and Pharmacy of Craiova, 200349 Craiova, Romania; 5 Department of Gastroenterology, University of Medicine and Pharmacy of Craiova, 200349 Craiova, Romania; 6 Department of Internal Medicine, Emergency Clinical County Hospital of Craiova, 200642 Craiova, Romania; 7 Department of Internal Medicine and Rheumatology, Dr. I Cantacuzino Hospital, 030167 Bucharest, Romania; anca-teodora.florescu@rez.umfcd.ro; 8 Department of Rheumatology, University of Medicine and Pharmacy of Craiova, 200349 Craiova, Romania; anca.musetescu@umfcv.ro

**Keywords:** immune checkpoint inhibitors, immune-related adverse events, arthritis, pneumonitis

## Abstract

**Simple Summary:**

Cancer therapy has evolved over the years, immunotherapy being the most used for untreatable malignant tumors. Immune checkpoint inhibitors decrease the ability of tumor cells to escape the immune system. Although immune checkpoint inhibitors have a significant impact in the treatment of cancer, they are associated with various adverse effects, mostly inflammation. The adverse events related to the immune system may affect basically every tissue in the human body, including the digestive tract, endocrine glands, liver, skin, cardiovascular, pulmonary and, also, rheumatic systems. In this review, we address the rheumatic immune-related adverse events related to immunotherapy by depicting the characteristics, diagnostic approach and treatment options.

**Abstract:**

The advent of immunotherapy has changed the management and therapeutic methods for a variety of malignant tumors in the last decade. Unlike traditional cytotoxic chemotherapy, which works by interfering with cancer cell growth via various pathways and stages of the cell cycle, cancer immunotherapy uses the immune system to reduce malignant cells’ ability to escape the immune system and combat cell proliferation. The widespread use of immune checkpoint inhibitors (ICIs) over the past 10 years has presented valuable information on the profiles of toxic adverse effects. The attenuation of T-lymphocyte inhibitory mechanisms by ICIs results in immune system hyperactivation, which, as expected, is associated with various adverse events defined by inflammation. These adverse events, known as immune-related adverse events (ir-AEs), may affect any type of tissue throughout the human body, which includes the digestive tract, endocrine glands, liver and skin, with reports of cardiovascular, pulmonary and rheumatic ir-AEs as well. The adverse events that arise from ICI therapy are both novel and unique compared to those of the conventional treatment options. Thus, they require a multidisciplinary approach and continuous updates on the diagnostic approach and management.

## 1. Introduction

The advent of immunotherapy has changed the management and therapeutic options methods for a variety of malignant tumors in the last decade. Unlike traditional cytotoxic chemotherapy, which works by interfering with cancer cell growth via various pathways and stages of the cell cycle, cancer immunotherapy uses the immune system to reduce malignant cells’ ability to escape the immune system and combat cell proliferation.

Immunotherapy may be divided into two types: passive and active. Immunoglobulins are considered to be a passive therapy method and bind to tumor-associated antigens, causing the immune system to clear them. The active type of immunotherapy works by activating the immune system to destroy tumor cells and target tumor antigens. Immune checkpoint inhibitor (ICI) therapy is considered an active category of immunotherapy [1,2].

Cell surface receptors such as cytotoxic T-lymphocyte antigen-4 (CTLA-4), programmed cell death protein 1 (PD-1) or programmed cell death ligand 1 (PD-L1) are targeted by ICIs, which cause tumor cells to be destroyed by the immune system.

Nowadays, the Food and Drug Administration (FDA) and the European Medicines Agency (EMA) have approved, after several clinical trials, the following immune checkpoint-blocking antibodies in cancer patients: anti-CTLA-4 (Ipilimumab); anti-PD-1 (Nivolumab, pembrolizumab and cemiplimab) and anti-PD-L1 (atezolizumab, avelumab and durvalumab) [3,4].

Ipilimumab was first approved for late-stage surgically unresectable melanoma in 2011 by the FDA based on the results of the MDX010-020 trial and is nowadays approved for the treatment of metastatic melanoma, advanced renal cell carcinoma and metastatic colorectal cancer, in combination with Nivolumab [5].

Nivolumab was first approved by the FDA in 2014 for late-stage unresectable/metastatic melanoma, following the outcome of the CheckMate-037 trial, which proved to have fewer toxic adverse events than Ipilimumab [6]. Currently, Nivolumab is approved for both small and non-small cell lung carcinoma, advanced renal cancer, Hodgkin’s lymphoma, recurrent or metastatic squamous cell cancer of the head and neck, urothelial cancer, colorectal cancer and hepatocellular carcinoma [7].

Pembrolizumab, a human IgG4k monoclonal antibody against PD-1, was first approved in 2014 after the clinical trial NCT01295827 for patients with metastatic melanoma who were refractory to CTLA-4 treatment [8]. After several clinical trials, pembrolizumab is currently approved for metastatic non-small- cell lung cancer, metastatic non-squamous non-small cell lung cancer, non-small cell lung cancer, recurrent or metastatic squamous cell cancer of the head and neck, Hodgkin’s lymphoma, urothelial cancer, third-line therapy for locally advanced or metastatic gastric/gastroesophageal junction/esophageal adenocarcinoma, mediastinal large B-cell lymphoma, hepatocellular carcinoma, Merkel cell carcinoma and metastatic renal cell carcinoma, with several levels of recommendations [9].

Cemiplimab was first approved by the FDA in 2018 for the treatment of locally advanced, metastatic cutaneous squamous cell carcinoma in patients who are not candidates for a curative surgery of radiation [10].

Avelumab received FDA approval in 2015 following the JAVELIN Merkel 200 trial for metastatic Merkel cell carcinoma with progressive disease or after chemotherapy and is nowadays also approved for urothelial carcinoma and renal cell carcinoma [11].

Durvalumab was approved in 2016 for the treatment of inoperable or metastatic urothelial bladder cancer and later approved for stage III non-small cell lung cancer [12].

Atezolizumab received its first approval in 2016 for locally advanced or metastatic urothelial carcinoma based on the IMvigor210 trial. Later on, it became approved following several clinical trials for metastatic non-small cell lung cancer, non-squamous non-small cell lung cancer, extensive-stage small-cell lung cancer and locally advanced or metastatic triple-negative breast cancer [3].

## 2. Mechanism of Development of Immune-Related Adverse Events

ICIs work by inhibiting the signal pathways that suppress tumor destruction mediated by T cells. T-lymphocytes normally play an important role in the cell-mediated clearance of cancer cells. Antigen-presenting cells are activated in the presence of a non-self cell, such as a tumor cell.

A tumor antigen is incorporated and presented by cells, such as dendritic cells or macrophages, via a major histocompatibility complex, which then binds to a T-cell receptor. T cells become activated in the presence of a costimulatory interaction, such as the one represented by the CD28 receptor, initiating a cascade of antitumor activity.

Various inhibitor receptors are upregulated during the T-cell activation process, acting as immune checkpoints to limit the overstimulation of the immune response. The CTLA-4 and PD-1 pathways, which have the normal function of suppressing the T-cell response and action, are two important pathways for ICIs [13].

Increased CTLA-4 binding, for example, in the presence of specific tumor cells, results in the competitive inhibition of costimulatory CD28 binding, resulting in decreased T-cell activation. Tumors may also express more PD-L1 receptors, resulting in decreased T-lymphocyte activity and tumor proliferation. Thus, it is thought that blocking the key components of either or both of these immune checkpoint pathways is responsible for the antitumoral activity of ICIs [14].

The CTLA-4 and PD-1 pathways are critical for immunological homeostasis, which helps prevent autoimmune disorders. Studies on knockout mice lacking these checkpoint molecules provide evidence for this involvement. As a result, rodents without CTLA-4 develop an aggressive immune-mediated disease characterized by the widespread activation of lymphocytes in the lymph nodes, spleen and thymus [15]. Since these mice have large amounts of immunoglobulin, the absence of renal involvement in knockout mice is intriguing. Anti-DNA and other antinuclear antibodies, which define systemic lupus erythematosus, a prototypical autoimmune illness with glomerulonephritis, are frequently related with increases in immunoglobulin synthesis in genetically modified mice [16].

In investigations of PD-1-deficient murine models, the relationship between autoimmunity and PD-1 was initially revealed. Mice missing PD-1 acquire a lupus-like illness with glomerulonephritis and IgG3 and C3 accumulation in the kidneys, depending on the strain. Although both CTLA-4 and PD-1-mutant mice suffer immune-mediated diseases, the symptoms are very different. Unlike CTLA-4 knockout mice, which die quickly, PD-1 knockout mice acquire illness gradually over the course of a year. Background genes appear to impact the development of autoimmunity when either checkpoint is inhibited [17,18].

Because the antigen specificity of T cells that mediate ir-AEs is unclear, research of the causes of these side effects have looked at the many T-cell repertoire traits as a link to the etiology. Ir-AEs may occur from the mobilization of vast numbers of T cells, some of which are autoreactive. The development of ir-AEs and the rise in clonal diversity may be linked. The lack of a link between T-cell receptor diversity and treatment impact might mean that autoreactive and antitumor cells are two separate populations [19].

While the purpose of ICI treatment is to enhance cytotoxic T cells, these drugs can have a direct or indirect effect on B cells. The number of circulating B cells in patients with advanced melanoma who received ICIs exhibited a substantial drop in the number of circulating B cells following treatment. Anti-PD-1 medication can promote B-cell autoimmunity initiated by anti-CTLA-4, and ir-AEs can develop over a longer period of time [20].

Future research directions need to define the relationship between antitumor and anti-self reactivity, develop biomarkers for predictions and determine new methods for the prevention and therapy of ir-AEs, especially in patients with pre-existing autoimmune diseases [21].

We wish to exemplify several rheumatic immune adverse effects that immune checkpoint inhibitor therapy encloses by presenting our clinical experience regarding a patient who developed rheumatic adverse events following a treatment with Nivolumab and Ipilimumab and, also, pneumonitis. Our aim is also to review the current diagnostic and therapeutic approaches regarding the adverse events related to joint, muscle and immune system involvement.

## 3. Clinical Experience—Case Report

We present the case of a 60-year-old male patient with non-small cell lung cancer and brain metastases (right hemiparesis) for which he underwent surgical treatment and radiotherapy, with pancreatic and adrenal metastases, under immunological treatment with immune checkpoint inhibitors, namely Nivolumab (anti-PD-1) 3 mg/kg IV every 2 weeks and Ipilimumab (anti-CTLA-4) 1 mg/kg IV every 6 weeks.

After 2 weeks of immunotherapy, the patient developed pain and swelling in the right elbow, associated with morning stiffness over 2 h and limited mobility, which was partially responsive to NSAIDs, and was admitted to Dr. I. Cantacuzino Hospital in Bucharest.

Laboratory tests revealed a biological inflammatory syndrome with a C-reactive protein (CRP) elevated more than five times the upper limit of normal, the erythrocyte sedimentation rate (ESR) within limits, complete blood count, C3 fraction of the complement, uric acid and alkaline phosphatase levels within the normal range, negative rheumatoid factor, anticitrullinated protein antibodies (ACPA) and ANA (antinuclear antibody) profile.

**Figure 1:**
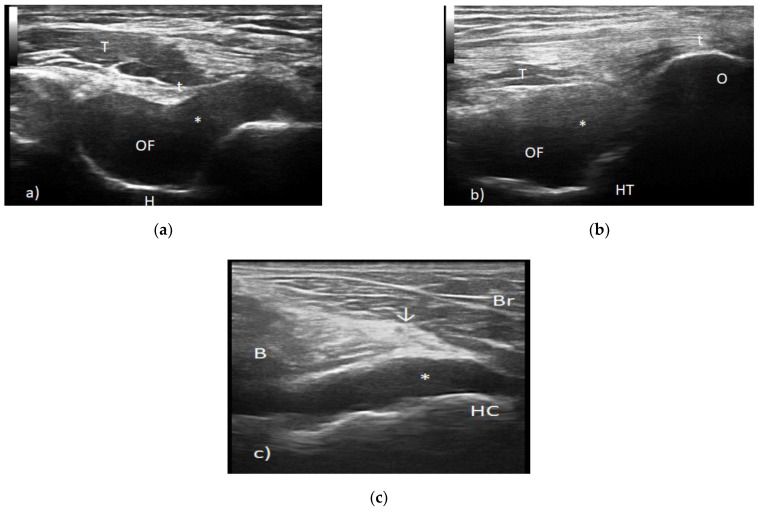
(**a**) Transverse scan posterior elbow. t—triceps tendon, T—triceps muscle, OF—olecranon fossa, *—synovial proliferation posterior joint recess and H—humerus. (**b**) Longitudinal scan posterior elbow. t—triceps tendon, T—triceps muscle, OF—olecranon fossa, *—synovial proliferation posterior joint recess and H—humeral trochlea. (**c**) Transverse view anterior elbow. B—brachialis muscle, Br—brachioradialis muscle, HC—humeral capitellum, ↓—radial nerve and *—anterior joint recess synovitis.

Elbow radiography showed degenerative changes with marginal osteophytes, while the musculoskeletal ultrasound revealed the distension of the articular capsule due to the presence of an anechoic/hypoechoic collection with the aspect of grade 2 synovial proliferation and the presence of a power Doppler signal (Figure 1).

Further, we considered the diagnosis of bone metastasis or arthritis in the context of immune therapy. A secondary tumor was ruled out by magnetic resonance imaging, so the diagnosis of arthritis in the context of ICIs was more likely.

We performed an intra-articular infiltration with corticosteroids under ultrasound guidance. The patient was also advised to follow the kinetic therapy protocol, leading to a favorable outcome with the remission of symptoms.

After 8 weeks of immunological therapy, the patient developed a skin rash on the scalp, with fast remission under topical corticosteroid administration, as directed by the dermatologist.

During all this time, the patient continued the oncological treatment according to the initial scheme.

**Figure 2:**
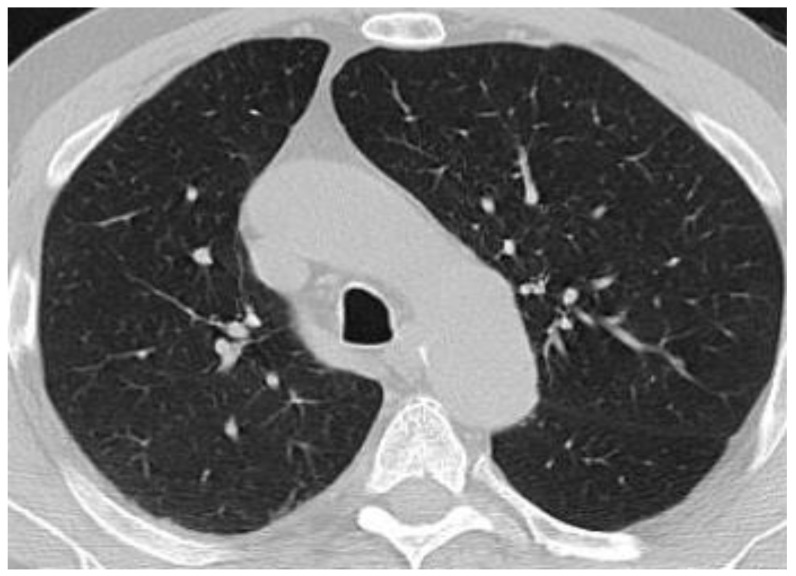
Computed tomography of the chest before immunotherapy.

**Figure 3:**
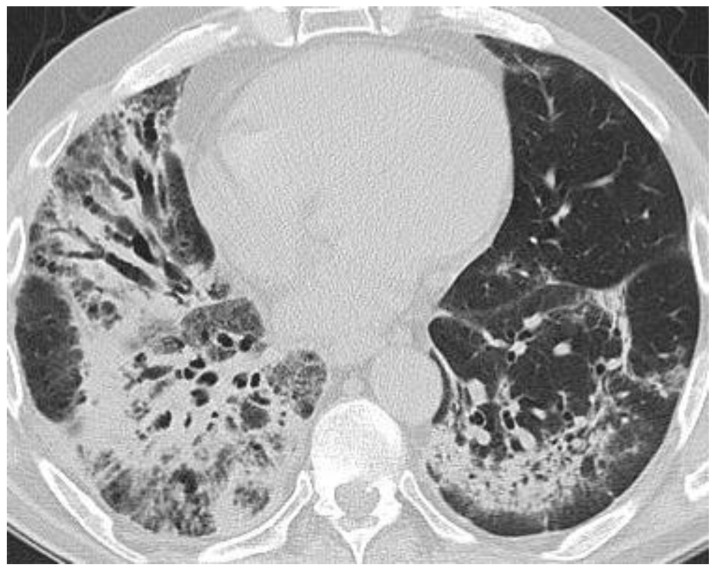
Chest computed tomography scan after 16 weeks of immunological treatment: ground-glass opacities in both lungs, more prominent in right lung.

At 16 weeks into oncological evaluation, the patient reported fatigue and rapidly progressing dyspnea. The patient was afebrile and denied chest pain or cough. Common pathogens of the lower inspiratory tract infections and, also, *Pneumocystis jirovecii*, *Legionella pneumophila, Chlamydia pneumoniae, Mycoplasma pneumoniae*, the flu virus and SARS-Co-V-2 infection were ruled out by performing sputum cultures, peripheral blood cultures, urinary antigen, bronchoalveolar lavage and nasopharynx swabs. Cardiac assessment (clinical exam, cardiac echography, NTproBNP) ruled out cardiac pulmonary edema. The bronchoalveolar lavage fluid showed lymphocyte alveolitis with an elevated percentage of lymphocytes of 49% but with negative cultures. A significant decrease in the forced vital capacity (17%) and diffusing capacity of carbon monoxide (DLCO) (23%) was observed during spirometry and plethysmography. Chest tomography revealed ground-glass opacities in both lungs, more prominent on the right side. We established the diagnosis of pneumonitis induced by immunotherapy (Figure 2 and Figure 3).

**Figure 4:**
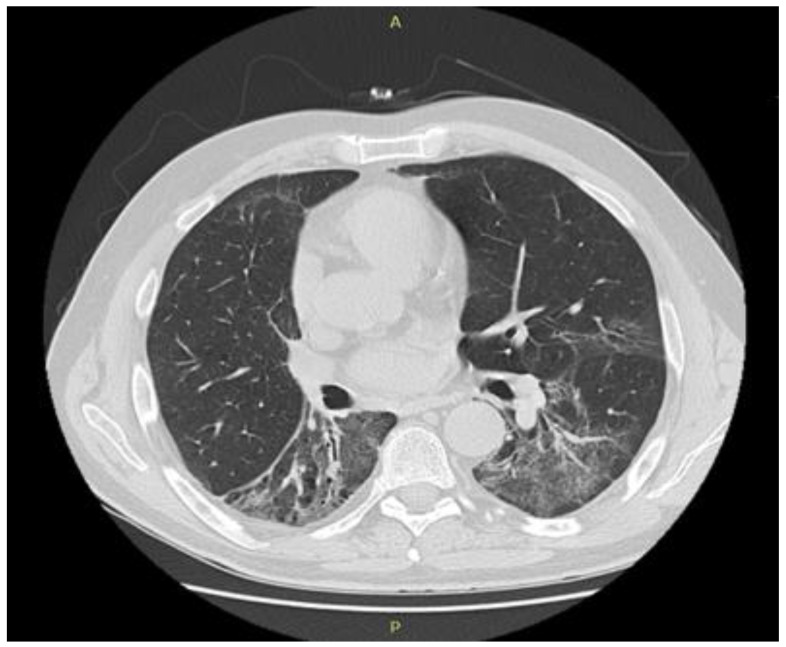
Chest computed tomography scan after 1 month of corticosteroid therapy: partial resolution of the interstitial infiltrates.

After the pneumological evaluation, corticosteroid therapy was initiated with dexamethasone 16 mg/day (the equivalent of 1mg/kg of prednisone per day) in tapering doses for 15 days, continued with prednisone 10 mg/day for one month, followed by the tapering of steroids for 3 months until exclusion. The evolution was favorable with the remission of pulmonary symptoms, the chest showing the complete remission of interstitial infiltrate tomography after 3 months of treatment (Figure 4).

To conclude, after 4 months of immunotherapy with Nivolumab and Ipilimumab, we observed the remission of brain, pancreatic and adrenal metastases and, also, a significant decrease in the size of the primary lung tumor. The patient developed numerous side effects (arthritis of the elbow, skin rash and interstitial pneumonitis), which had a favorable outcome and complete response to corticosteroid therapy.

Analyzing the literature, as in our case, in the context of combination therapy with Nivolumab and Ipilimumab, one of the most common Ir-AEs is a skin rash, which occurs in the first weeks of immunotherapy (3–6 weeks). These complications are usually mild and reversible within a few weeks under topical corticosteroid administration [22].

The incidence of musculoskeletal-adverse effects is 10% in combination therapy patients. Although several studies observed that arthralgia/arthritis occurs later after the initiation of immunological therapy, our patient developed elbow arthritis after only 2 weeks. The symptoms subsided under intra-articular steroids [23].

Interstitial pneumonitis is a potentially life-threatening ir-AE that occurs in approximately 10% of patients in combination therapy. According to Sarah Chuzi et al., checkpoint inhibitor-related pneumonitis (CIP) occurs after an average of 2.5 months after the initiation of immune therapy. In our case, the patient developed grade II CIP after 16 weeks and was successfully treated with steroids, according to the guidelines [24].

Our patient did not require a more aggressive treatment, such as the additional immunosuppression with csDMARDs or bDMARDs for elbow arthritis, skin rash or interstitial pneumonitis, emphasizing the importance of an early diagnosis and prompt treatment of ir-AEs. The management of the ir-AEs was guided, taking into consideration the patient’s wishes and, also, the judgement of both the rheumatologist and oncologist. The patient continued the oncological treatment according to the initial scheme throughout this period. The favorable evolution from an oncological point of view supports the data from the literature, according to which, the patients who developed rheumatic ir-AEs had higher tumor response rates [25].

## 4. Rheumatic Immune-Related Adverse Events

The widespread use of ICIs over the past 10 years has presented valuable information on the profile of toxic adverse effects. The attenuation of T-lymphocyte inhibitory mechanisms by ICIs results in immune system hyperactivation, which, as expected, is associated with various adverse events defined by inflammation. These adverse events, known as immune-related adverse events (ir-AEs), may affect any type of tissue throughout the human body, which includes the digestive tract, endocrine glands, liver and skin, with reports of cardiovascular, pulmonary and rheumatic ir-AEs as well.

In the oncology and rheumatology literature, three major clinical phenotypes induced by cancer immunotherapy have been described: arthritis, myositis and polymyalgia-like syndrome. Additionally, there have been reported cases of sicca syndrome, scleroderma, vasculitis and sarcoidosis [26,27].

### 4.1. Inflammatory Arthritis

Joint involvement, such as arthralgias/arthritis, secondary to ICI administration, has been described in several case series and cohort studies. Arthralgias were reported to occur in a wide range of 1–40% of patients treated with ICIs during clinical trials, while inflammatory arthritis does not seem to exceed 5–7%. The median time from ICI initiation to symptom onset appears to be less than three months. Joint involvement has been evidenced after only one dose of ICIs but can also occur up to 2 years after immunotherapy, sometimes becoming chronic despite the cessation of ICIs [28].

Monoarthritis, most commonly involving the shoulder, as well as oligoarthritis and polyarthritis, have been reported. These patients were priorly treated with anti-PD1/PDL1 or combination ICIs.

Patterns resembling rheumatoid arthritis (RA) have been described involving metacarpophalangeal, proximal interphalangeal and wrist and knee joints, sometimes even causing erosive disease. However, the rheumatoid factor and anticitrullinated protein antibodies (ACPAs) are often negative, erosions and tendon involvement appear more prominent early in the course of the disease and there is no female predominance. Approximately 20% of the patients fulfilled the classification criteria for RA [29].

There have been reported cases of asymmetric oligoarthritis, inflammatory back pain, enthesitis and dactylitis mimicking spondylarthritis. Nevertheless, concomitant psoriatic lesions have rarely been described, and human leukocyte antigen (HLA)-B27 positivity has not been evidenced. A reactive arthritis-like pattern with sterile urethritis, conjunctivitis and oligoarthritis has also been described in a few cases [30,31].

Recently, recurrent pseudo-gout flares at 7–10 days after each Nivolumab infusion have been recently reported. Case reports of remitting seronegative symmetrical polyarthritis with pitting edema (RS3PE) syndrome have also been described in the literature.

Structural abnormalities such as synovitis, tenosynovitis, dactylitis, enthesitis, bone erosions and the presence of a power Doppler signal have been noted when using a musculoskeletal ultrasound and magnetic resonance imaging. In some cases, inflammatory changes have not been depicted [32,33,34].

### 4.2. Polymyalgia Rheumatica-Like Syndrome

Classic polymyalgia rheumatica (PMR) is defined by proximal muscle tenderness, typically affecting the pelvic and scapular joints and muscles. Common complaints include morning stiffness and fatigue. The absence of typical RA autoantibodies, age over 50 years old and an increase in inflammatory markers are critical characteristics for its diagnosis [35].

A similar syndrome has been reported in patients taking ICIs, with a prevalence of 2% to 3% in different case series. It is worth noting that 25% of the cases failed to meet the provisional 2012 classification criteria for the PMR requirements. Atypical characteristics were discovered, such as the involvement of other joints (mostly knees and hands), the absence of elevated inflammatory markers and the prevalence of aggressive cases that were resistant to standard corticosteroid therapy [36].

For both anti-CTLA4 and anti-PD1/PDL1 inhibitors, the majority of cases of this PMR-like syndrome tend to occur early, within the first months after ICI therapy initiation. It is of great importance to look for signs of temporal arteritis, such as headache and vision loss, in the presence of a PMR-like syndrome [37].

### 4.3. Myositis

Idiopathic inflammatory myopathies (IIM) are a group of pathologies defined by proximal muscle weakness with elevated muscle destruction enzymes and specific electromyographic and histopathological findings. Classic dermatomyositis (DM) has a clear link to cancer; thus, the name paraneoplastic myositis is often used in these cases.

Myositis is becoming more widely known as an ir-AE (ir-myositis), but it still affects just about 1% of patients exposed to ICIs. Furthermore, as compared to the idiopathic types of the disorder, ICI-induced myositis has atypical characteristics and a high mortality rate, indicating that it is the most severe musculoskeletal ir-AE [38,39].

In most cases, distinguishing ir-myositis is easy; patients presenting with rapidly developing weakness of the proximal muscles groups and elevated muscle destruction enzymes. Distal, axial and oculobulbar weakness, dysphagia, diaphragmatic weakness and rash are some of the other symptoms. Some patients attribute their symptoms to the cancerous pathology and associated complications, thus delaying the diagnosis [40].

Ir-myositis has been proven to associate with myocarditis and myasthenia gravis more frequently than classical myopathies.

Touat et al. defined a specific number of characteristics that define ir-myositis, as follows: (1) early and severe onset of manifestations within 2 months of ICIs therapy, (2) pelvic and scapular-girdle weakness in association with myalgias, as well as axial and oculomotor weakness, (3) marked creatinine kinase elevations and myopathic alterations in electromyographic studies, (4) no myositis-specific and anti-acetylcholine receptor antibodies, (5) necrosis and inflammation revealed at the histopathological exam and (6) a strong response to ICI cessation with or without corticosteroid therapy [41].

### 4.4. Vasculitis

There have been reports of vasculitic ir-AE (ir-vasculitis) affecting large, medium and small vessels. Vasculitis of the large vessels, such as giant cell arteritis (GCA) and isolated aortitis, and vasculitis of the nervous system, such as primary angiitis of the central nervous system and, also, peripheral nerve angiitis, were the most common. There have been reports of cases of granulomatosis with polyangiitis (GPA), eosinophilic granulomatosis with polyangiitis (EGPA) and cryoglobulinemic vasculitis. There have also been reports of cutaneous granulomatous and leukocytoclastic vasculitides. Vasculitis affecting single organs such as the retina, testicles and uterus have also been described. Nevertheless, vasculitides are more frequent in melanoma patients treated with ICIs [42].

Several other cases of vasculitis have been recorded, including case reports of acral vasculitis that resulted in digital necrosis and distal digit amputation despite aggressive immunosuppression [28].

The clinical, biological and histopathological manifestations of ir-vasculitis and idiopathic vasculitides appear to be nearly identical. The preponderance of large-vessel vasculitis is also consistent with the malfunction of the PD-1/PD-L1 pathway.

Autoantibodies are rarely found in patients with ICI-induced vasculitis, and the key serological finding is an increase in inflammatory markers. In the majority of patients, the discontinuation of ICI and immunosuppression with large doses of steroids resulted in the partial or full resolution of the clinical manifestations [43].

### 4.5. Sarcoidosis/Sarcoid-Like Syndrome

A number of cases of sarcoidosis or sarcoid-like reactions induced by ICIs have been reported in the recent literature, with anti-CTLA4 or anti-PD1 being used more frequently than anti-PDL1 or combination therapy. Although the pathogenic mechanism of sarcoidosis induced by ICIs is unknown, it is worth noting that a decreased CTLA4 expression in Tregs and Th17 lymphocytes has been identified in patients with sarcoidosis [44].

ICI-induced sarcoidosis can manifest as cutaneous sarcoidosis such as nodules; rash in almost half of patients or can be systemic with lymphadenopathy; lung involvement or neurological, ocular and articular involvement. For ICI-induced sarcoidosis, there are no specific biological findings. The levels of serum angiotensin-converting enzyme (SACE) can be high or low.

Melanoma is the most common underlying malignancy linked to sarcoid-like reactions, with a slight female preponderance. Sarcoid-like reactions may appear anywhere between three weeks to two years after starting ICI, with the lungs and skin being the most frequently affected organs [45].

In terms of imaging, patients may have parenchymal lung CT changes, such as ground-glass opacities, in addition to mediastinal or hilar lymphadenopathy. This is important in clinical practice, because sarcoid-like reactions may be mistaken for disease progression. Biopsies of lymph nodes taken in these cases to rule out cancer recurrence or progression often revealed non-necrotizing granulomatous inflammation [46].

### 4.6. Sicca/Sjögren’s Syndrome

Immune checkpoint imbalances can cause autoreactive cells to become activated and proliferate, which can lead to Sjögren’s syndrome (SS). CTLA4 polymorphisms have been linked to disease susceptibility and the development of autoantibodies.

CTLA4 deletion in murine Tregs led to sialadenitis, and PDL-1 has been shown to protect nondiabetic obese mice from developing SS. These findings point to a close correlation between immune checkpoint dysfunction and the development of sicca symptoms. In clinical trials, sicca has been identified as an ir-AE with an incidence rate ranging from 1.2% to 24.2% [47].

The median time from treatment initiation to ir-AE was 3.8 months. After starting ICI therapy, dry mouth symptoms emerged after a median of 70 days. Antinuclear antibodies (ANA), extractable nuclear antigens (ENA) and rheumatoid factor were all negative in the majority of patients. In contrast with the histological profile of idiopathic SS, the salivary glands proved to have a predominant infiltrate consisting of T cells with acinar destruction [48].

In comparison to SS, which is almost exclusively a female disorder, patients with sicca symptoms induced by ICIs are more likely to be male. Dry mouth is the most common symptom and ocular and oral dryness seldom coexist. A low incidence of abnormal ocular tests is seen in patients with ICI-induced sicca [49].

### 4.7. Scleroderma

In the literature, only a few cases of scleroderma skin reactions or systemic scleroderma after ICI therapy have been identified. Anti-PD1 therapy was previously administered to the patients, and they presented with skin thickening, rare lung involvement and only one reported case of new onset Raynaud’s phenomenon. Specific serum antibodies were negative in all reported cases [50].

### 4.8. Systemic Lupus Erythematosus

Immune checkpoint dysregulation has been linked to the development of systemic lupus erythematosus (SLE). PD1 and CTLA4 gene polymorphisms in humans, as well as deficiencies in animal models, have been linked to lupus manifestations. SLE induced by the administration of ICIs is rare. There have also been reports of cutaneous lupus, lupus nephritis, neuro-lupus and Jaccoud arthropathy. The median time of symptom development was almost 200 days, while the mean age was 61, with a female-to-male ratio of 1.6:1 [51].

The several types of syndromes presented may be a consequence of immune checkpoint inhibitor therapy but may also be a sign of progressive cancer, a paraneoplastic syndrome or a rheumatic disease occurring in concurrence, making the differentiation between the possible etiologies somehow difficult at times. However, the presence of specific antibodies, markers of the inflammatory syndrome, imaging studies, the response to NSAIDs and corticosteroids or to the discontinuation of cancer treatment may guide clinicians to a specific etiology [52].

### 4.9. Management of Rheumatic Ir-AEs

Regarding the management of ir-AEs, EULAR has formulated several points to take into consideration, such as:

- Local and/or systemic corticosteroids should be taken into consideration for ir-AEs if the symptomatic treatment is ineffective, depending on the severity of the disease. The dose regimen and administration route should be chosen based on the clinical entity and activity. If the patient’s condition improves, systemic corticosteroids should be tapered to the lowest dosage possible in order to manage the symptoms.
  Symptomatic treatments such as NSAIDs and/or analgesics should be taken into consideration for mild-to-moderate rheumatic manifestation. Corticosteroids may be used locally—intra-articular for mono/oligoarticular involvement—while system corticosteroids are recommended for polyarticular involvement and, also, other systemic manifestations. The dose ranges from a 20 to 60-mg/day prednisone equivalent depending on the affected system, and the doses should be tapered to the lowest dose possible.
- In patients who do not respond to an appropriate dose of glucocorticoids or who need glucocorticoid sparing therapy, conventional synthetic disease-modifying anti-rheumatic drugs (csDMARDs) should be considered.
  The most frequently used csDMARD remains methotrexate, followed by hydroxychloroquine and sulphasalazine. There have also been reports of cyclophosphamide use in vasculitis.
- Patients with serious ir-AEs, as well as those who do not have the desired response to csDMARD, should consider biological (b) DMARDs, the preferred treatment option for inflammatory arthritis being TNF or IL-6 inhibitors. The most frequently used TNF inhibitor was infliximab in a series of cases with refractory arthritis.
- The decision to stop or continue ICIs should be focused on the severity of ir-AEs, the magnitude of the required immunosuppressive therapy dosage, the tumor response and the length of administration, as well as the prospective oncology treatment follow-up.
  Currently, the option of withholding or continuing cancer treatment is variable from hospital to hospital. The need for standardized care in ir-AEs is imminent nowadays, prospective trials being able to clarify the optimal immunosuppressive regimens.
- Myositis is a severe rheumatic condition. Clinicians should consider stopping immunotherapy. Large doses of corticosteroids—1 to 2 mg/kg/day—intravenous immunoglobulins and/or plasmapheresis should be taken into consideration in the presence of severe manifestations that are life-threatening (dysphagia, dysarthria, dysphonia, dyspnea and myocarditis). Immunotherapy withdrawal is always essential. Additionally, there have been reports of DMARD usage, such as mycophenolate mofetil, methotrexate, azathioprine and hydroxychloroquine.
- The use of ICIs should not be ruled out because of a pre-existing autoimmune rheumatic and/or systemic disorder. The initial immunosuppressive regimen should be held to the lowest possible dosage (if possible, below 10-mg prednisone per day for corticosteroids). Many patients, however, can experience a flare-up of their underlying condition, as well as immune-related side effects, requiring corticosteroids or DMARDs.
- There is no need to screen every patient for the existence of autoantibodies before beginning cancer immunotherapy. A full rheumatological assessment should be performed in the case of unexplained rheumatic symptoms [53].

The management of ir-AEs should be multidisciplinary. Since there are evidence-based guidelines for the treatment of rheumatic ir-AEs, the treatment options should be discussed between the patient, rheumatologist and oncologist [54].

## 5. ICI Therapy-Related Pneumonitis

Pneumonitis related to the administration of ICI therapy is a severe ir-AE associated with high morbidity and a cessation of immunotherapy and may even be a cause of mortality. The estimated incidence of ICI-related pneumonitis is between 3% and 6%, with higher incidence rates among the patients with non-small lung cell carcinoma and renal carcinoma. Pneumonitis is more prominent to develop in patients receiving combination therapy rather than monotherapy [55].

The presenting symptoms of pneumonitis may be variable and nonspecific, ranging from asymptomatic (almost one-third of patients at onset) to dyspnea, cough and even fever and chest pain.

According to the National Cancer Institute CTCAE Pneumonitis Grading System, ICI therapy-related pneumonitis may be graded as follows: grade 1—asymptomatic; grade 2—symptomatic, medical intervention indicated; grade 3—severe symptoms, limited self-care ADL, oxygen indicated; grade 4—life-threatening respiratory compromise, urgent intervention indicated and grade 5—death [56].

The imaging techniques, especially a computer tomography scan of the chest, may depict certain pneumonitis patterns, such as: organizing pneumonia (OP), hypersensitivity pneumonitis (HP), nonspecific interstitial pneumonia (NSIP), bronchiolitis, acute interstitial pneumonitis-adult respiratory distress syndrome (AIP-ARDS) and recall radiation [57].

The American Society of Clinical Oncology has formulated Clinical Practice Guidelines for the management of ICI-related pneumonitis. According to the guide, grade 1 pneumonitis should be treated by withholding ICI therapy if there is radiographic evidence of progressive lesions. Grade 1 should be treated as grade 2 pneumonitis if there is a lack of improvement after follow-up. Grade 2 pneumonitis should benefit from withholding ICI therapy until resolution to grade 1 or less, followed by the administration co-corticosteroid therapy tapered over 4–6 weeks. In the case of grade 3 and 4 pneumonitis, ICI therapy should be permanently discontinued, followed by the administration of empirical antibiotics or intravenous methylprednisolone therapy. Additionally, if there is no improvement, infliximab, mycophenolate mofetil or intravenous immunoglobulin therapy may be required [58].

## 6. Conclusions

Immune checkpoint inhibitor therapies have become of great use in oncology during the last years, being used as first- and second-line therapeutic agents in multiple malignancies. The adverse events that arise from ICI therapy are both novel and unique compared to those of the conventional treatment options. Thus, they require a multidisciplinary approach and continuous updates on the diagnostic process and management.

## Data Availability

Not applicable.
